# Mapping the AI life sciences landscape in Greece: a bibliometric comparison with global patterns

**DOI:** 10.1038/s41598-026-56107-2

**Published:** 2026-06-03

**Authors:** Eleni Adamidi, Serafeim Chatzopoulos, Alexandros C. Dimopoulos, Anastasia Krithara, Anastasios Nentidis, Nikos Pechlivanis, Fotis Psomopoulos, Thanasis Vergoulis, Kleanthis Vichos

**Affiliations:** 1 Athena Research Center, IMSI, Aigialias 19 & Chalepa, Marousi, Athens, 15125 Greece; 2 PSYCHNOW LLC, 737 N. Michigan Avenue, Suite 2240, Chicago, IL 60611 USA; 3 Institute for Fundamental Biomedical Science, Biomedical Sciences Research Center “Alexander Fleming”, Vari, Greece; 4 Department of Informatics and Telematics, School of Digital Technology, Harokopio University, Athens, Greece; 5 Institute of Informatics and Telecommunications, National Centre for Scientific Research (NCSR) “Demokritos”, Agia Paraskevi, 15310 Greece; 6 Institute of Applied Biosciences, Centre for Research and Technology Hellas, 6th km Charilaou-Thermis rd, Thermi, Thessaloniki, 57001 Greece

**Keywords:** Computational biology and bioinformatics, Health care, Mathematics and computing, Scientific community

## Abstract

Artificial intelligence is increasingly used in Life Sciences, though the pace and direction of adoption varies widely across countries. To map the Greek landscape, we performed a data‑driven analysis of 916,824 AI-related life-science papers harvested from OpenAlex and PubMed. We tagged each publication with Medical Subject Headings (MeSH) and compared topic frequencies between articles linked to at least one Greek institution and the rest of the world. Greek‑affiliated outputs are disproportionately concentrated under the theme of methodology and algorithm‑development, whereas the global corpus is dominated by disease‑focused, organism‑centered and clinical applications. Statistical contrasts across three MeSH hierarchy levels exposed clear national strengths in machine learning techniques and analytical tools, alongside under‑representation in translational, patient‑centred research. Overall this study combines bibliometric evidence with community perspectives and provides a comprehensive overview of AI activity in Life Sciences in Greece, highlighting potential thematic strengths and gaps.

## Introduction

Artificial Intelligence (AI) has rapidly evolved over the past decade, becoming one of the most influential technological advancements across scientific fields. The impact is particularly evident in research and innovation, where it has reshaped methodologies, enabled new discoveries, and offered solutions to previously complex problems. AI methods are increasingly used in Life Sciences to assist the analysis of biological data facilitating the extraction of valuable insights and the discovery of latent patterns. Their applications span a wide spectrum, including predictive modeling in genomics and systems biology^1,2^, image analysis for diagnostics^1^, optimization of experimental workflows^3^, and, more recently, the use of Large Language Models (LLMs) for clinical data interpretation^4,5^.

As the integration of AI in Life Sciences becomes increasingly widespread, a growing number of international initiatives have emerged to explore the infrastructural, ethical, and research implications of this transformation, both across and within individual communities or countries. Recent bibliometric studies have mapped the geographic distribution of AI-enabled life-science research and documented regional disparities in AI-related biomedical publishing, underscoring the value of country- and region-level analyses^6,7^. Such country-level research profile comparisons have been used in prior scientometric and bibliometric mapping work^8,9^. One such complementary effort is the Report by UKRI^10^ on digital research infrastructure requirements for AI, which provides a national-level perspective on the challenges and opportunities associated with AI adoption across disciplines. It identifies critical needs in terms of skills, data management, compute infrastructure, and interdisciplinary collaboration. Inspired by the scope of this UK-based initiative, we sought to carry out a similar effort tailored to the context of Greece.

Greece has a growing research ecosystem in Life Sciences and computational technologies, yet there is still limited systematic understanding of how AI is currently being applied in this domain at a national level. Our study aims to fill this gap by mapping the intersection between AI and Life Sciences in Greece. This mapping is timely, as recent investments in national research infrastructure and the emergence of collaborative initiatives (e.g., ELIXIR-GR^11^ have begun to support computationally intensive work in biology, biomedicine, and related fields. Community-oriented efforts within Greece also reflect this growth, including recent syntheses of domain-specific bioinformatics activity^12^.

To investigate the state of AI activity in Life Sciences in Greece, we carried out a data-driven analysis using bibliographic datasets from OpenAlex^13^ and PubMed^14^. This involved retrieving a large collection of publications at the intersection of Life Sciences and AI-related fields (e.g., machine learning, natural language processing) and enriching them with valuable metadata, including institutional affiliations, topics (OpenAlex concepts), and subject headings (MeSH terms).

The bibliometric analysis enabled us to capture both perspectives from the Greek community and evidence of research activity in the AI–Life Sciences interface. Our findings aim to offer insights into thematic patterns, institutional expertise, and training landscapes in Greece, and to highlight how they compare with broader international patterns. Moreover, the analysis supports a discussion of national research priorities and infrastructural investments needed to facilitate further AI integration in the Life Sciences.

## Results

The overarching analysis process consisted of consecutive data retrieval, annotation and filtering steps, followed by a comparative statistical analysis between the selected groups. OpenAlex served as the primary source of bibliographic metadata. By linking each publication’s DOI to its corresponding MeSH in PubMed, we compiled a richly annotated corpus of articles positioned at the intersection of Medicine and Biology, and further associated with topics such as Natural Language Processing, Artificial Intelligence, or Machine Learning (see Methods). The final dataset was then divided into two groups, “Greece” and “World”, depending on whether a publication listed at least one institution located in Greece among its affiliations. This division allowed us to explore how the thematic focus of AI–Life Sciences research in Greece aligns with – or diverges from – the global landscape.

### Comparative overview of Greek and global AI–life sciences research

To understand the thematic structure of AI Life Sciences research, we focused on the main topics of these publications, based on their Medical Subject Headings (MeSH)^15^ annotations. To build a comprehensive literature dataset we combined literature records with detailed affiliation information. This required matching publications that included both MeSH descriptors and author affiliations. Specifically, out of all the 916,824 publications initially retrieved from OpenAlex, 712,749 publications were annotated with MeSH descriptors and affiliation information, and out of these, 3,514 included at least one Greek affiliation. These MeSH annotations, representing the main topics of each publication, were used in order to investigate which topics had higher or lower frequency in publications from Greece compared to publications from the rest of the world, as described in Methods. The data source for MeSH annotations was the 2024 version of the PubMed Baseline Repository^14^, and MeSH annotations were available for about 78% of the publications, as PubMed has specific inclusion criteria related to the scope and quality of the publication journal^16^.

### Thematic and statistical differences across MeSH categories

#### General overview Greece vs. world (High-level MeSH categories)

At the top level, comparing MeSH Categories, which are the roots of the MeSH polyhierarchy, we observed that the frequency of publications from Greece was lower in the categories of “Anthropology, Education, Sociology, and Social Phenomena” and “Psychiatry and Psychology”, and “Disciplines and Occupations”, which was statistically significant, as shown in Table 1. The remaining MeSH categories had smaller insignificant differences as seen in Table 1. To note, the significance symbols are defined as follows (used consistently across the tables): p > 0.05 ”ns” (not significant), *p* ≤ 0.05 ”*”, *p* ≤  0.01 "**”, *p* ≤  0.001 "***”, and *p* ≤ 0.001 "****”.

**Table 1:** Comparing the frequency of publications from Greece against publications from the rest of the world at the level of MeSH categories (Statistical analysis). Ranked by estimate.

MeSH category (level 0)	Estimate	Stat. sign.
Technology, industry, and agriculture	0.014	ns
Chemicals and drugs	0.003	ns
Information science	0.003	ns
Phenomena and processes	0.001	ns
Named groups	0.000	ns
Anatomy	0.000	ns
Humanities	-0.003	ns
Diseases	-0.006	ns
Analytical, diagnostic and therapeutic techniques, and equipment	-0.006	ns
Organisms	-0.008	ns
Health care	-0.008	ns
Georgaphics	-0.016	ns
Anthropology, education, sociology, and social phenomena	-0.024	**
Psychiatry and psychology	-0.024	**
Disciplines and occupations	-0.025	**

#### General overview Greece vs. the world (mid-level MeSH categories)

Moving one level below, that is, to direct descendants of the MeSH Categories, we encounter 113 “MeSH subcategories” of level 1. Of them, only 16 had a statistically significant (i.e. not ns), as shown in Table 2. For most of them (~ 69%), the difference was negative, revealing some topics where the relative frequency of publications from Greece was found to be lower than the rest of the world. For five subcategories, however, the difference was positive, indicating that these topics are more frequent in publications from Greece than the rest of the world, proportionally. Table 2 also reveals that differences observed at one hierarchical level are not necessarily distributed evenly into subordinate levels, where differences of varying magnitude and significance may be observed, even in the opposite direction. The MeSH subcategories “Urogenital Diseases”, “Nutritional and Metabolic Diseases”, and “Neoplasms””, for instance, have a positive difference, which is also significant, despite being included in the broader category “Diseases” that has a slightly negative difference, overall, which is not substantial. At the same time, the “Diseases” category includes four subcategories with a significant negative difference. A similar situation holds for the “Diagnosis” subcategory, which presents a positive difference in frequency, despite belonging to a Category without a substantial difference overall.

**Table 2:** Comparing the frequency of publications from Greece against publications from the rest of the world at the level of MeSH subcategories (MeSH descriptors of level 1). Only cases that are significant (i.e., not ns) at level 1 are presented, ranked by estimate. The respective MeSH category is shown for completeness.

MeSH category (level 0)	MeSH subcategory (level 1)	Estimate	Stat. sign.
Diseases	Urogenital diseases	0.044	**
Diseases	Nutritional and metabolic diseases	0.037	*
Diseases	Neoplasms	0.035	***
Phenomena and processes	Mathematical concepts	0.022	*
Analytical, diagnostic and therapeutic techniques, and equipment	Diagnosis	0.013	*
Anthropology, education, sociology, and social phenomena	Social sciences	-0.024	*
Diseases	Cardiovascular diseases	-0.025	*
Psychiatry and psychology	Psychological phenomena	-0.027	**
Health care	Population characteristics	-0.028	*
Psychiatry and psychology	Behavior and behavior mechanisms	-0.030	**
Health care	Health care economics and organizations	-0.034	*
Anthropology, education, sociology, and social phenomena	Education	-0.039	*
Diseases	Stomatognathic diseases	-0.053	*
Disciplines and occupations	Health occupations	-0.061	****
Diseases	Infections	-0.065	**
Diseases	Respiratory tract diseases	-0.069	**

#### Selected cases in comparison

Moving to descriptors at level 2 of the MeSH hierarchy, that is, direct descendants of MeSH subcategories, we can observe that an overall frequency difference that is significant in a higher-level category is often explained by respective differences in only a few descriptors at a lower level. Table 3, for instance, reveals that the lower frequency of the “Psychiatry and Psychology” category in Greece is mainly explained by substantially lower frequencies in just five level-2 descriptors. The remaining fifty level-2 descriptors that belong to “Psychiatry and Psychology” present only insignificant differences (i.e., ns).

**Table 3:** Comparing the frequency of publications from Greece against publications from the rest of the world, considering MeSH descriptors of level 2 that belong to MeSH Category “Psychiatry and Psychology”. Only selected cases that are significant (i.e., not ns) at level 2 are presented, grouped by Subcategory, and ranked by estimate.

MeSH category (level 0)	MeSH subcategory (level 1)	MeSH descriptor (level 2)	Estimate	Stat. sign.
Psychiatry and psychology	Behavior and behavior mechanisms	Behavior	-0.032	*
		Psychology, social	-0.05	**
		Attitude	-0.044	*
		…	…	…
	Behavioral disciplines and activities	Psychology, social	-0.05	**
		…	…	…
	Psychological phenomena	Mental processes	-0.04	**
		…	…	…
…	…	…	…	…

Similarly, the positive difference in the “Technology, Industry, and Agriculture” category, though observed as insignificant at level 0, is mainly explained by respective significant positive differences in level-2 descriptors, as shown in Table 4, with the tens of their remaining sibling descriptors at the same level presenting insignificant frequency differences between Greece and the rest of the world. This may suggest that smaller frequency decreases in several other level-2 descriptors inside this category may counteract the effect of the decreased frequency of these two descriptors.

**Table 4:** Comparing the frequency of publications from Greece against publications from the rest of the world, considering MeSH descriptors of level 2 that belong to MeSH Category “Technology, Industry, and Agriculture”. Only selected cases that are significant at level 2 are presented, grouped by Subcategory, and ranked by estimate.

MeSH category (level 0)	MeSH subcategory (level 1)	MeSH descriptor (level 2)	Estimate	Stat. sign.
Technology, industry, and agriculture	Technology, industry, and agriculture	Engineering	0.078	**
		Alloys	0.263	*
		…	…	…
	…	…	…	…

Finally, considering this level of the polyhierarchy, the phenomenon of conflicting frequency differences inside the same category becomes even clearer. For instance, this happens in the category “Phenomena and Processes” with three level-2 descriptors presenting positive differences and three presenting negative, as shown in Table 5. This phenomenon is also observed in five more categories, namely “Anatomy”, “Chemicals and Drugs”, “Diseases”, “Health Care”, and “Analytical, Diagnostic and Therapeutic Techniques, and Equipment”.

**Table 5:** Comparing the frequency of publications from Greece against publications from the rest of the world, considering MeSH descriptors of level 2 that belong to MeSH Category “Phenomena and Processes”. Only selected cases that are significant at level 2 are presented, grouped by Subcategory, and ranked by estimate.

MeSH category (level 0)	MeSH subcategory (level 1)	MeSH descriptor (level 2)	Estimate	Stat. sign.
Phenomena and processes	Cell physiological phenomena	Cell count	-0.153	*
		Radiation tolerance	0.202	*
		…	…	…
	Genetic phenomena	Selection, genetic	-0.124	*
		Mutagenesis	-0.162	*
		…	…	…
	Mathematical concepts	Neural networks, computer	0.070	**
		Algorithms	0.027	*
		…	…	…
	…	…	…	…

#### Comparison between the two time periods (2000–2010 and 2011–2020)

Finally, it is worth reviewing if there are any major patterns in the comparison between the two periods, i.e. 2000–2010 and 2011–2020. As presented in Table 6, in the first decade, most top-level categories have lower frequencies in Greece than in the world, which is also statistically significant for about half of them. Only “Technology, Industry, and Agriculture” has a higher frequency, which is not statistically significant. In the second decade, interestingly, several categories have a higher frequency in Greece than in the world, which is, however, statistically significant only for one of them, namely “Chemicals and Drugs”. The category “Anthropology, Education, Sociology, and Social Phenomena” is the only one with a significantly lower frequency in this decade, with all other categories having insignificant frequency differences. The three categories that present significantly lower frequency in Greece when considering the overall 20-year-long period have a consistently lower frequency in each of the two periods, which, however, is only significant in one of them. Overall, frequency shifts between these two decades seem to justify the low significance levels in most differences observed for top-level categories in the overall analysis.

**Table 6:** Comparing the frequency of publications from Greece against publications from the rest of the world at the level of MeSH categories (Statistical analysis), considering two time periods. Ranked by overall estimate.

MeSH category (level 0)	Estimate overall	Stat. Sign. overall	Estimate 1st dec.	Stat. Sign. 1st dec.	Estimate 2nd dec.	Stat. Sign. 2nd dec.
Technology, industry, and agriculture	0.014	ns	0.005	ns	-0.011	ns
Chemicals and drugs	0.003	ns	-0.040	ns	0.049	**
Information science	0.003	ns	-0.012	ns	0.012	ns
Phenomena and processes	0.001	ns	-0.030	*	0.016	ns
Named groups	0.000	ns	-0.026	ns	0.016	ns
Anatomy	0.000	ns	-0.040	ns	0.000	ns
Humanities	-0.003	ns	-0.138	**	0.044	ns
Diseases	-0.006	ns	-0.063	****	0.010	ns
Analytical, diagnostic and therapeutic …	-0.006	ns	-0.040	**	0.005	ns
Organisms	-0.008	ns	-0.043	***	0.003	ns
Health care	-0.008	ns	-0.044	**	0.000	ns
Georgaphics	-0.016	ns	-0.028	ns	-0.010	ns
Anthropology, education, sociology, and …	-0.024	**	-0.037	ns	-0.060	**
Psychiatry and psychology	-0.024	**	-0.048	*	-0.019	ns
Disciplines and occupations	-0.025	**	-0.111	****	-0.023	ns

## Discussion

The findings of the systematic analysis of the literature highlight a distinct thematic divergence between Greece and global patterns in the use of AI techniques within the Life Sciences. In Greece, the focus appears to be on the foundational development of tools, methods, and machine learning algorithms. A hypothesis that could potentially explain this finding is that it is likely driven by computer science and engineering departments within Greece, in contrast to international efforts that may be led by life sciences departments (where the main driver is basic biomedical research). This divide, if confirmed, could shape the nature of AI-related work and priorities across each context.

A closer look at selected MeSH subcategories further illustrates these differences in research orientation. For example, “Mathematical Concepts” and “Diagnosis” appear with higher relative frequency in Greek-affiliated publications, pointing to a strong national footprint in the development of computational methodologies and diagnostic tools. Conversely, topics such as “Behavior and Behavior Mechanisms” and “Psychological Phenomena” are markedly underrepresented compared to the global corpus, suggesting a lower degree of engagement with clinically embedded or patient-centered AI applications. Interestingly, within the broader “Diseases” category, Greek research shows a relatively higher focus on “Urogenital Diseases”, “Nutritional and Metabolic Diseases”, and “Neoplasms”, whereas internationally the emphasis is distributed more evenly across multiple disease areas, including cardiovascular, stomatognathic, and respiratory tract diseases. This pattern indicates that national strengths align with core algorithmic development and selected biomedical domains, while translational and behavioral health applications remain comparatively underexplored.

It should be noted that, based on the (arguably broad and not systematic)overview of the available MSc programs, this perspective is also evident, as overall, the identified programs aim to equip students with interdisciplinary skills combining data science, informatics, and engineering to address complex challenges in biomedical research, healthcare innovation, and translational medicine. Another methodological limitation is that our country assignment follows a binary affiliation rule (≥ 1 Greek affiliation), which does not capture fractional contributions in multi-country collaborations and may therefore bias thematic contrasts, particularly for papers where the Greek contribution is small. Future work could evaluate fractional/weighted attribution and collaboration-stratified sensitivity analyses to assess the robustness of the observed differences. However, this work is focussed primarily on identifying the main patterns of presence/absence, rather than the quantified presence across a number of thematics. As such, doing a fractional counting would not offer any additional insights (or make any difference) to the produced results of this work.

Finally, it is worth mentioning that the findings of this work are reinforced by independent policy documents. A key example is the 2022 Policy Support Facility (PSF) Final Review report to Greece for policies developing research infrastructures and the R&I ecosystem^17^, in which it is stated that “[.] Greece also has a very strong background in informatics and computer science and given the growing visibility of artificial intelligence (and the importance this has in the Greek strategic planning)[.]”, as well as the fact that AI can blend well with bioinformatics applications. Both of these points support the findings of our analysis in the unique strengths of the Greek scientific community in the use of AI techniques within the Life Sciences.

## Methods

Data retrieval, annotation, and filtering were carried out in successive stages, followed by a systematic statistical analysis between the selected groups. All code that has been developed specifically for this analysis, is openly and publicly available under an MIT license in the GitHub repository https://github.com/BiodataAnalysisGroup/Elixir-GR-ML.

### MeSH thesaurus

The MeSH thesaurus is a controlled vocabulary developed and maintained by the National Library of Medicine (NLM). The basic entity in MeSH is the MeSH descriptor, which is a set of related terms. MeSH descriptors are hierarchically organized into sixteen predefined groups, named top-level MeSH categories^18^, that represent general topics such as anatomy, organisms, and diseases^19^, so that each MeSH descriptor represents a narrower topic of at least one broader MeSH descriptor or MeSH category. MeSH descriptors are widely used in biomedical knowledge systems, primarily for the semantic indexing of scientific literature. In the MEDLINE part of PubMed^20^, in particular, publications are annotated with topic labels primarily using MeSH descriptors. A process that used to be done by human indexers and has gradually transitioned to automation^21^.

### Data sources and retrieval

OpenAlex^13^ categorizes the vast world of scholarly research through a sophisticated hierarchical framework. At its core, the system utilizes a taxonomy of approximately 65,000 concepts, which trace their lineage back to the Microsoft Academic Graph. This structure is organized into 19 broad, root-level categories that branch out into five distinct levels of depth, allowing for both general and granular classification. To ensure consistency and interoperability, every concept in OpenAlex is directly linked to Wikidata; consequently, each one is assigned a unique Wikidata ID as its standard identifier. The actual process of tagging scholarly works is handled by an open-source, automated classifier. By analyzing the titles and abstracts of research papers, this model evaluates the content to determine which concepts apply. This automated approach is remarkably effective, successfully assigning at least one relevant concept to roughly 85% of the works within the OpenAlex database.

OpenAlex served as the principal source for bibliographic information for this study. As one of the most comprehensive Scholarly Knowledge Graphs (SKGs), OpenAlex provides extensive metadata for research products (e.g. publication and datasets) as well as their relationship with other research-related entities (e.g., organizations, scientific topics).

In this study, we focused on publications explicitly linked with the OpenAlex top-level concepts of “**Medicine**” and “**Biology**”, and at the same time categorized under “**Natural Language Processing**”, “**Artificial Intelligence**”, or “**Machine Learning**”. For each retrieved publication, we collected rich metadata including title, authors, affiliations, DOI, PMID, and topic annotations (the first two levels of OpenAlex concepts hierarchy).

The entire data acquisition process was implemented using Python, leveraging the October 2024 OpenAlex snapshot^22^ to retrieve, filter and transform relevant metadata into a convenient format for further analysis.

Subsequently, each DOI was linked with its corresponding Medical Subject Heading (MeSH) terms as cataloged in PubMed. MeSH terms constitute a regulated vocabulary employed by the National Library of Medicine for the indexing and organization of biological content. They are arranged in a hierarchical framework comprising many levels, from broad categories (e.g., “Diseases” or “Analytical, Diagnostic, and Therapeutic Techniques”) to more specific, detailed descriptions.

All data are available at 10.5281/zenodo.17415352 and 10.5281/zenodo.17407809.

### Data curation (pre-processing)

Multiple preprocessing steps were implemented to ensure the robustness of the study, all carried out using R (R-4.4.0). The dataset was divided into two categories: articles associated with Greek institutions (group “Greece”) and all other publications (group “World”). A publication was assigned to the “Greece” group if it listed at least one institution located in Greece.

Next, we calculated the frequency of each MeSH term for both groups over the period 2000–2020. Since MeSH terms are organized hierarchically across multiple levels; this study focused on terms from the first three levels. To mitigate potential bias due to differences in annual publication volumes, relative abundances were calculated by normalizing MeSH term frequencies against the total number of publications for each year. Finally, z-scoring was applied where appropriate (i.e., in heatmaps) to highlight the differences between the two groups.

All code used for this preprocessing is available at https://github.com/BiodataAnalysisGroup/Elixir-GR-ML.

### Statistical analysis

Generalised linear models were fitted independently for each MeSH term, including year, group, and their interaction as predictors, in order to find topics with significant differences between the two groups (world vs. Greece). The results were then compiled into a color-coded, multi-level table, linking each level 2 topic to its corresponding level 1 and level 0 ones. This allowed us to identify patterns across the MeSH hierarchy.

### Community assessment

#### Mapping of training programs in Greece

Additionally, in order to provide some additional context and insights, we performed a review of available courses (under-/post-graduate level) in Greece on the AI/LS intersection. To this end, we compiled and shared a list of master’s degree programs offered in Greece that focus on the intersection of machine learning or artificial intelligence and biology. We collected 12 courses from 8 public universities and research organizations in Greece offering in person, online or hybrid master courses (list can be found here: 10.5281/zenodo.20041753).

These postgraduate programmes focus on advanced topics at the intersection of data science, bioinformatics, biomedical engineering, medical informatics, and AI. A systematic overview of the provided descriptions and available content across these programmes, showed that the key themes include:

- Data science & big data analytics applied to health, life sciences, and bioinformatics.
- Bioinformatics and computational biology, including NGS data analysis and use of AI technologies (e.g., machine learning, deep learning, neural networks, knowledge graphs).
- Precision and personalized medicine, emphasizing molecular and genomic data for individualized therapy.
- Biomedical engineering, covering areas such as biofabrication, molecular diagnostics, imaging, and medical information systems to support precision medicine.
- Health statistics and data analytics, training graduates to analyze and interpret health data for research, public health, and clinical applications.
- Digital health and AI in healthcare, focusing on the development and use of advanced digital tools, ICT, and AI technologies in health systems.

It should be noted that some programmes also include modules on computational medicine, simulation, computer security, and the application of ICT in education and healthcare.

## Data Availability

All data are available at [https://doi.org/10.5281/zenodo.17415352](https:/doi.org/10.5281/zenodo.17415352) and [https://doi.org/10.5281/zenodo.17407809](https:/doi.org/10.5281/zenodo.17407809) .
